# Evaluation of a modified Bunnell suture technique with sequential tensioning for tension-offloading in Achilles tendon rupture repair: a case series and description of the surgical technique

**DOI:** 10.1093/jscr/rjag111

**Published:** 2026-02-27

**Authors:** Abdulla Aljawder, Noor Jaragh, Osama Zeidan, Maryam Almahmeed

**Affiliations:** Orthopaedic Surgery Department, King Hamad University Hospital, Building 2435, Road 2835, Block 228, PO Box 24343, Busaiteen, Bahrain; School of Medicine, Royal College of Surgeons in Ireland – Medical University of Bahrain (RCSI-MUB), Building 2441, Road 2835, Block 228, Busaiteen, PO Box 15503, Adliya, Bahrain; School of Medicine, Royal College of Surgeons in Ireland – Medical University of Bahrain (RCSI-MUB), Building 2441, Road 2835, Block 228, Busaiteen, PO Box 15503, Adliya, Bahrain; Orthopaedic Surgery Department, King Hamad University Hospital, Building 2435, Road 2835, Block 228, PO Box 24343, Busaiteen, Bahrain

**Keywords:** Achilles tendon rupture, modified Bunnell suture, sequential tensioning, limited-open surgery, early rehabilitation

## Abstract

Acute Achilles tendon (ATR) rupture is a common and potentially disabling injury, particularly in active young adults. Whilst operative repair offers lower re-rupture rates, traditional open techniques carry risks of wound complications and delayed mobilization. We describe a modified limited-open repair technique incorporating sequential suture tensioning to enhance functional recovery and enable early weight-bearing. Our case series presents five patients (aged 33–46) with acute ATR managed using a lateralised limited-open approach, preserving the paratenon. A modified Bunnell suture technique with FiberTape was applied using three crisscrossed loops, each tensioned sequentially from distal to proximal. Postoperative care included early mobilization and physiotherapy. ATRS scores ranged from 75 to 99 (mean: 88.8). The technique demonstrated biomechanical stability, early recovery, and good outcomes.

## Introduction

Acute Achilles tendon rupture (ATR) is a common injury amongst young active adults between the ages of 30 and 50, particularly men. [1]. Acute ATR usually occurs due to a singular high-load impact injury with no warning symptoms and with distinct ankle trauma (forceful dorsiflexion) [2].

ATR is commonly seen in football, tennis, jumping, badminton, and running [2]. Sporting activities play an essential role in the development of the pathology of ATRs, especially if improper training sessions are being performed [3].

Histologically, findings include high vascularity, hypercellularity near the rupture site, and collagen disarrangement. This leads to decreased tensile strength which predisposes patients to rupture [4]. Ruptures are most commonly total, a partial rupture is relatively rare. Other factors that contribute to decreased tensile strength of the tendon include pathological conditions like arteriosclerosis, hyperthyroidism, and diabetes [5].

Whilst ATR could be managed conservatively or surgically, operative repair is often preferred in active patients due to lower re-rupture rates and faster return to function compared to nonoperative options like functional bracing or casting [6]. However, traditional open techniques, though effective, carry higher risks of infection, nerve injury, and delayed mobilization [7].

Over the past decade, mini-open and percutaneous approaches have gained popularity for minimizing soft tissue trauma and promoting earlier weight-bearing [6]. Different suturing techniques were also suggested to ensure proper restoration of the length of the tendon and tension, both remaining critical to optimizing functional recovery and biomechanical outcomes. Systems like the Percutaneous Achilles Repair System (PARS) facilitate suture passage through small incisions but remain limited by high cost and reliance on specialized equipment [8, 9].

Many protocols still favour prolonged immobilization, contributing to muscle atrophy, stiffness, and delayed recovery [10]. Whilst early mobilization has shown promise following strong repairs, consensus is lacking on which techniques best enable safe early weight-bearing without increasing re-rupture risk [11].

Our case series, presenting five patients, reports a modified Bunnell suturing technique with sequential tensioning. This study aims to evaluate its feasibility, safety, and early functional outcomes, including recovery timelines, weight-bearing, and validated ATRS scores compared to traditional immobilization protocols.

## Description of surgical technique

**Figure 1:**
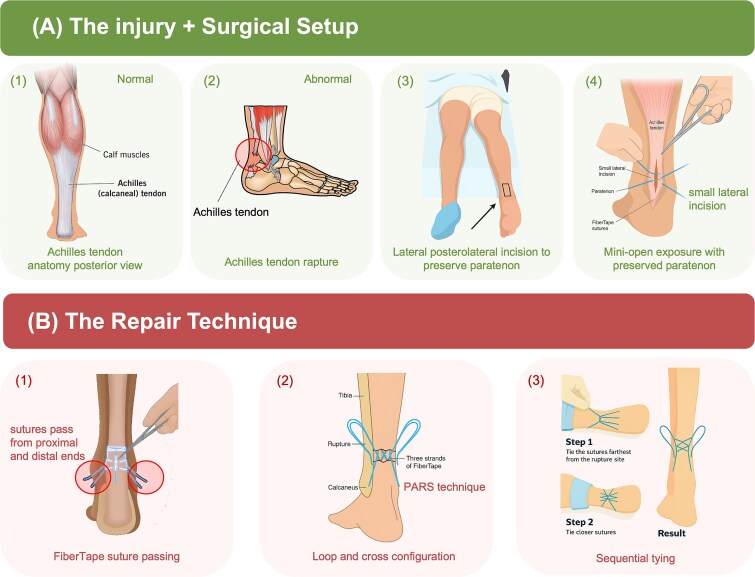
Illustration of the modified limited-open Achilles tendon repair technique. (A) The injury and surgical setup. (B) The repair technique.

In this case series, we propose several key principles for the repair of acute ATRs. The procedure begins with a limited lateral open incision (mini-hybrid approach), positioned away from the midline of the tendon. Careful dissection is performed to avoid stripping or incising the paratenon sheath during exposure. Preserving the paratenon is critical for maintaining vascularity and facilitating biological healing at the rupture site; hence, this approach is used to minimize the risk of injury to the paratenon or the tendon substance itself (Fig. 1A).

The tendon repair utilizes a modified PARS technique, performed without the use of a jig, allowing for minimally invasive yet precise suture placement. The repair employs FiberTape, a strong, non-absorbable, 2 mm braided suture material. Each FiberTape is looped through the tendon in a crisscrossed configuration: the suture is passed through the tendon (Fig. 1B1), looped at one end, crossed over, looped again, and crossed once more (Fig. 1B2). A total of three FiberTapes are used, creating six robust suture strands within the tendon.

A unique aspect of the technique is the sequential tensioning strategy. The strands are tied sequentially, beginning from the points farthest from the rupture site and progressing towards the centre. This allows for controlled tension offloading across the repair zone (Fig. 1B3). The final construct is mechanically robust and effectively distributes load across a broad section of the tendon, enabling healing with minimal direct stress at the rupture site.

## Case presentation

This case series includes five patients, ranging from 33 to 46 years old, of whom four are males and one is a female, who sustained acute ATR. All cases were managed through a modified Bunnell technique using a limited-open lateral approach, conducted by the same surgeon following an identical protocol. None had significant comorbidities or previous Achilles tendinopathy. Postoperative care was standardized. Table 1 indicates that all patients commenced partial weight-bearing 2–4 weeks postoperatively, utilized the ankle stabilizing orthosis (ASO) brace for 3 weeks, and experienced no reported complications or re-ruptures. The duration for returning to sports ranged significantly from 2 months to 1 year, presumably affected by personal objectives and activity levels. ATRS scores varied from 75 to 99, with a mean of 88.8, signifying elevated satisfaction and substantial functional recovery across the series. No wound complications, infections, or re-ruptures were observed.

**Table 1:** Summary of functional recovery patient-reported outcomes after Achilles tendon repair.

	Age (years)	Gender	Mechanism of injury	Time to WB (weeks)	ACO brace (duration)	ATRS (0–100)	Return to sports / ADL	Complications
Patient 1	42	Male	Traumatic	3 weeks	3 weeks	99	2 months	No
Patient 2	35	Male	Activity	2 weeks	3 weeks	87	4 months	No
Patient 3	47	Female	Not specified	3 weeks	3 weeks	90	8 months	No
Patient 4	38	Male	Not specified	4 weeks	3 weeks	93	1 year	No
Patient 5	33	Male	Sports-related rupture	3 weeks	3 weeks	75	Not yet	No

**Figure 2:**
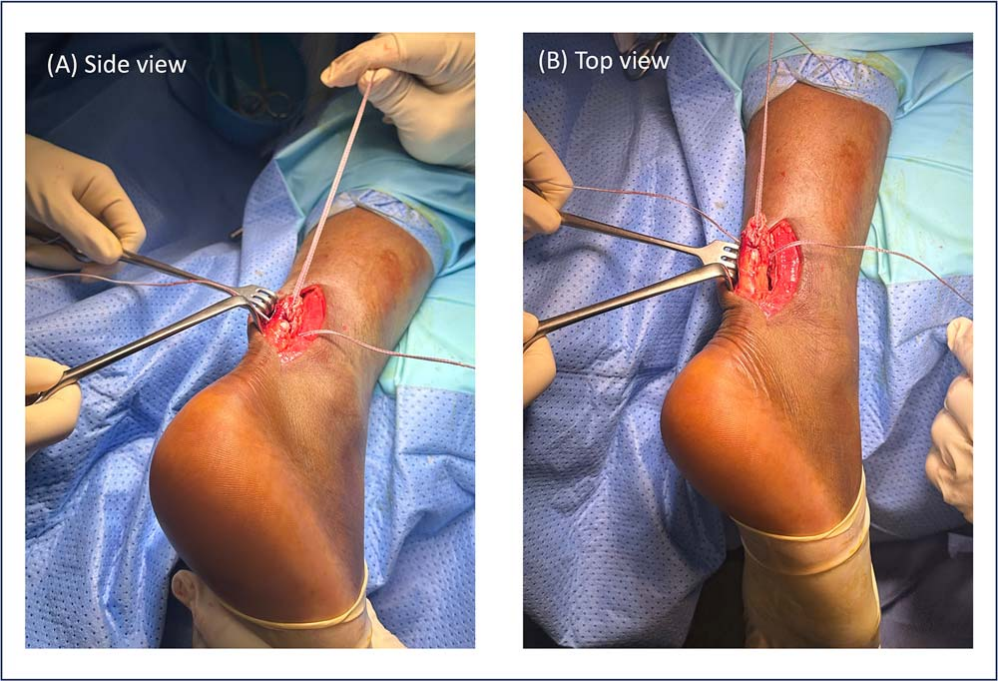
Intraoperative views of Achilles tendon repair in Patient 1 using the modified Bunnell technique. (A) Side view (A) and top view (B) demonstrate three crisscrossed FiberTape loops applied with sequential tensioning.

Patient 1, with the ATRS score of 99, returned to sports within 2 months post-operatively. Figure 2 demonstrates intraoperative images of this patient, showcasing the suture repair technique using three crisscrossed FiberTape loops in a sequential tensioning strategy. With no jigs or commercial systems used, it reduces dependency on costly equipment.

Patient 2, a 35-year-old male, began partial weight-bearing at 2 weeks, which is earlier than the other patients, but as reported by his ATRS score, he tolerated it well without complications. He returned to sports after 4 months. His ATRS score, slightly below the series average at 87, indicates full confidence in walking and light jogging, as well as a score of 8 for walking on uneven surfaces. Patients 3 and 4 showed ATRS scores of 90 and 93, respectively, with no functional limitations or postoperative complications. These outcomes support the feasibility of early weight-bearing in patients following secure repair and careful monitoring of rehabilitation.

**Figure 3:**
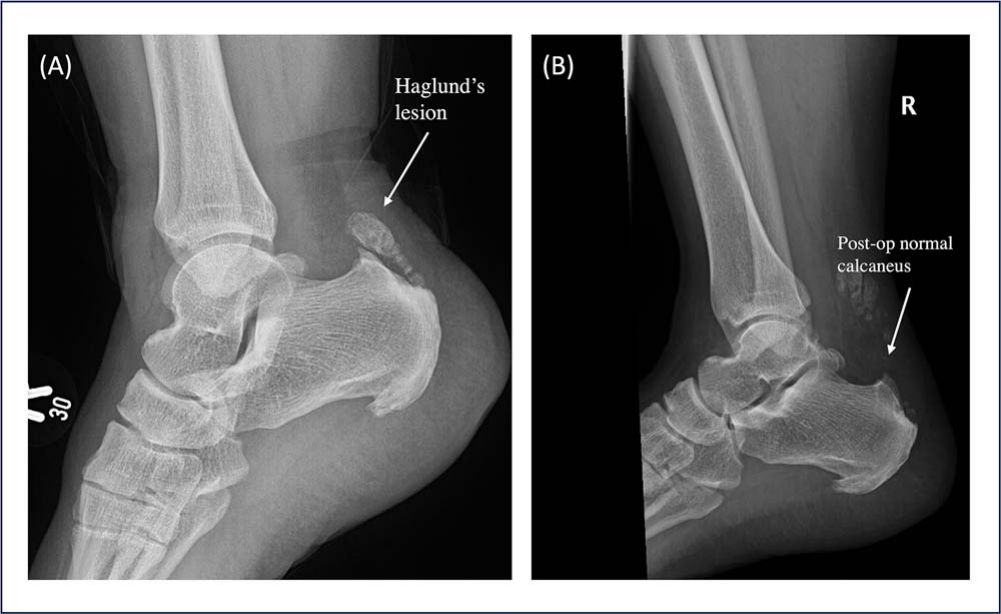
Pre- and postoperative lateral ankle X-ray of Patient 5. (A) Pre-operative shows a prominent Haglund’s lesion before surgery. (B) The postoperative image demonstrates a normalized calcaneal contour following debridement and Achilles tendon repair, suggesting adequate resection and restoration of the tendon insertion profile.

**Figure 4:**
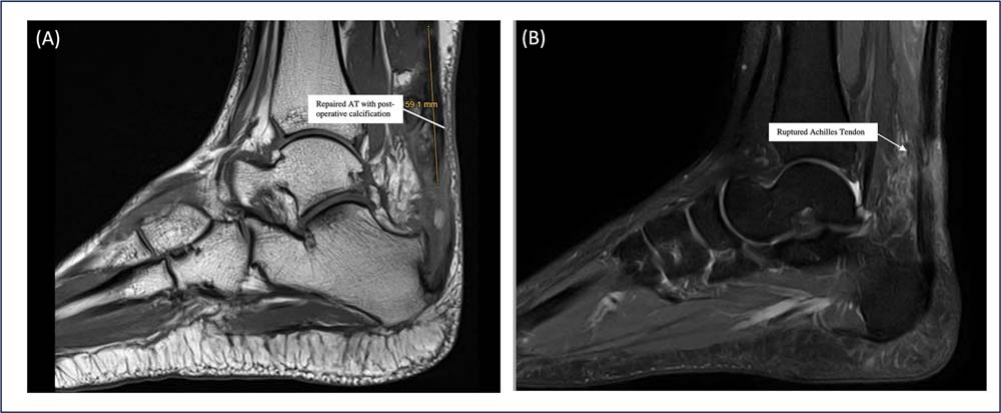
Sagittal MRI sequences of Patient 5 showing calcific changes and tendon status. (A) MRI highlights a 6 mm postoperative calcification near the Achilles tendon insertion site. (B) MRI confirms intact Achilles tendon continuity with no evidence of re-tear.

Patient 5 is a 46-year-old female. The patient sustained an ankle twisting injury leading to acute ATR. She has a pre-existing Haglund deformity (Fig. 3A) and developed postoperative calcification near the tendon insertion, which is 6 mm in length. However, no functional limitations or discomfort during dorsiflexion were felt clinically (Fig. 4); this might be a mild complication secondary to local irritation of the tendon or altered healing due to the presence of a bony prominence. She underwent right Achilles tendon repair with flexor hallucis longus (FHL) transport and Y lengthening. She began weight-bearing at 3 weeks following the same protocol as other patients. However, at the final follow up the patient had not yet returned to sports and reported an ATRS score of 75, the lowest amongst the patients in the series. This indicates that there are ongoing limitations in function or confidence. Whilst the patient’s surgical and post-operative course was uneventful, this may reflect delayed tendon remodelling, reduced strength, pain or psychosocial factors such as fear of re-injury.

**Figure 5:**
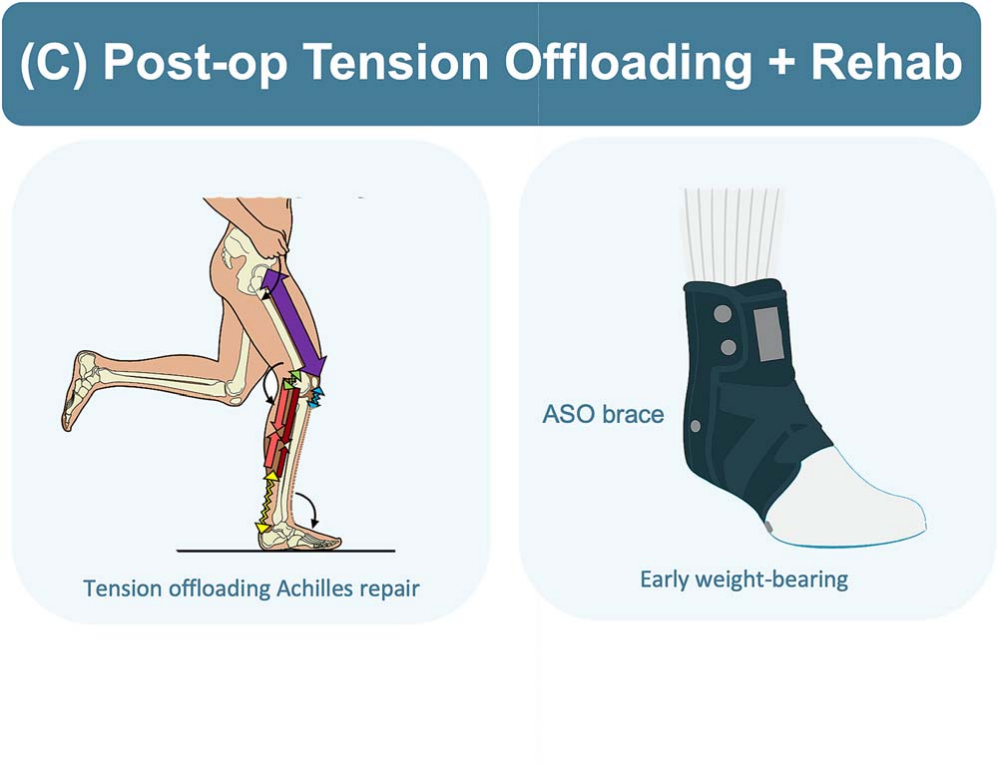
Illustrations showing postoperative rehabilitation and tension offloading strategy.

All patients started on an ASO brace at 3 weeks postoperatively. The ASO brace was selected due to its ability to stabilize and balance the ankle and for functional mobility, facilitating an early return to partial weight-bearing. Physiotherapy and rehabilitation were initiated concurrently, focusing on a controlled range of motion exercises. This postoperative strategy, combined with the robustness of the sequentially tensioned suturing technique, is designed to offload tension from the rupture site and support safe early weight-bearing (Fig. 5). No patients suffered any complications such as re-rupture, supporting the feasibility of this protocol.

## Discussion

Our case series presents a cost-effective, jig-free, limited-open approach to acute ATR repair using FiberTape and sequential tensioning. Our findings support the safety and functional efficacy of this technique, especially for active adults requiring rehabilitation and early return to sports. With no re-ruptures, zero wound complications, and an average ATRS of 88.8 within 12 months, this technique demonstrates favourable short-term outcomes.

### Functional and clinical outcomes

The early functional recovery observed in our patients underscores the potential benefits of this modified repair approach. Four out of five patients achieved ATRS scores above 87, reflecting minimal limitations in pain, strength, and activity. These results surpass average ATRS outcomes reported in the literature (between 75 and 85) for early rehabilitation protocols [12]. Furthermore, 80% of our patients resumed partial weight-bearing within 3 weeks, a notable improvement over traditional immobilization timelines, which often delay loading for 6–8 weeks [11]. All patients returned to activity or sports within 2–12 months, except one patient who stated that they preferred to stop doing sports due to fear of re-injury, with no reported re-ruptures or delayed wound healing. These outcomes suggest that early weight-bearing may be safely facilitated when supported by a strong and biologically preserved construct.

### Sequential tensioning as the biomechanical rationale

A distinguishing feature of our technique is sequential suture tensioning, which involves tying FiberTape limbs in a distal-to-proximal sequence, addressing a recognized limitation in tendon repair through the concentration of stress at the central rupture point, which is typically the most vulnerable to elongation or gap formation. By progressively offloading tension towards the periphery, sequential tensioning promotes a more balanced force distribution across the repair site. Biomechanical studies on tendon and ligament constructs support this strategy, showing that tension-sharing configurations reduce central gapping by up to 42% avoiding poor healing or failure and enhance repair stiffness by 30%–50% [9, 11]. Furthermore, FiberTape, with a load-to-failure exceeding 250 Newtons (N), significantly outperforms conventional sutures like FiberWire (almost 140 N), especially in multi-strand arrangements like the ones in our modified technique [8]. This enhanced mechanical integrity likely contributes to our patients’ early functional gains and absence of re-rupture.

### Biological considerations: paratenon preservation

**Figure 6:**
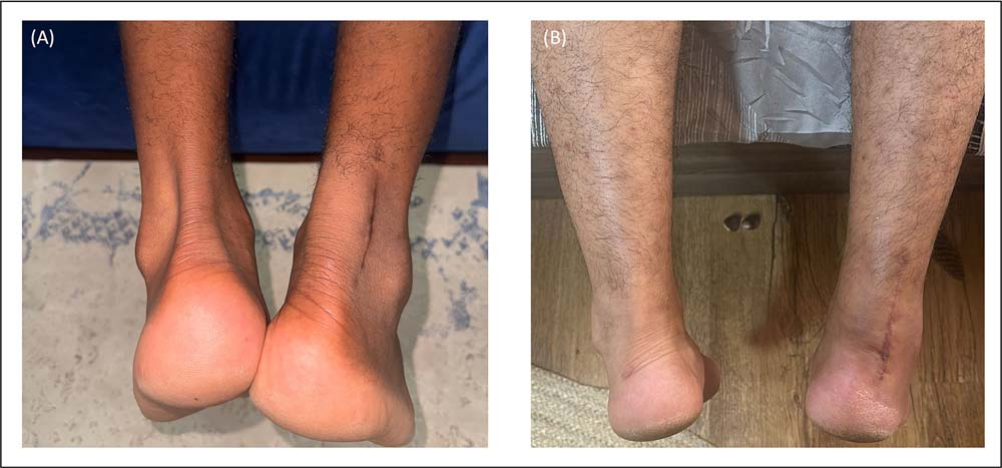
Post-operative images of healed ATR scars demonstrating minimal scarring, well-approximated skin edges, and absence of any wound complications.

Biological healing is equally vital to repair success, particularly in a structure as vascular-dependent as the Achilles tendon. The dissection we did via a lateralized, limited-open incision allowed us to preserve the paratenon, a vascular-rich sheath critical for tendon healing, fibroblast migration, and collagen remodelling. Studies have demonstrated that maintaining the integrity of the paratenon improves tendon strength by up to 40% at 6 weeks post-repair and accelerates neovascularization and collagen alignment [7]. By avoiding midline incisions and reducing unnecessary soft tissue disruption, our technique respects these biological healing mechanisms, which likely contributed to our zero wound complication rate and smooth postoperative recoveries. Refer to Fig. 6 for images of post-operative ATR surgical sites. Figure 6A was taken 10 months post-operatively, whilst Fig. 6B was captured 3 months post-operatively.

### Rehabilitation implications and advantages

Traditional protocols for ATR repair often favour prolonged immobilization, which can contribute to muscle atrophy and delayed gait restoration [10]. In contrast, our patients progressed rapidly through rehabilitation, with ASO brace use discontinued by week six. Early loading in a stable construct, as provided by our method, has been associated with faster return to normal gait (average 4 weeks vs. 8–12 weeks) and improved patient satisfaction (12–14). The high average ATRS and consistent patient-reported outcomes in our series affirm the functional viability of this accelerated protocol.

### Clinical strengths

**Figure 7:**
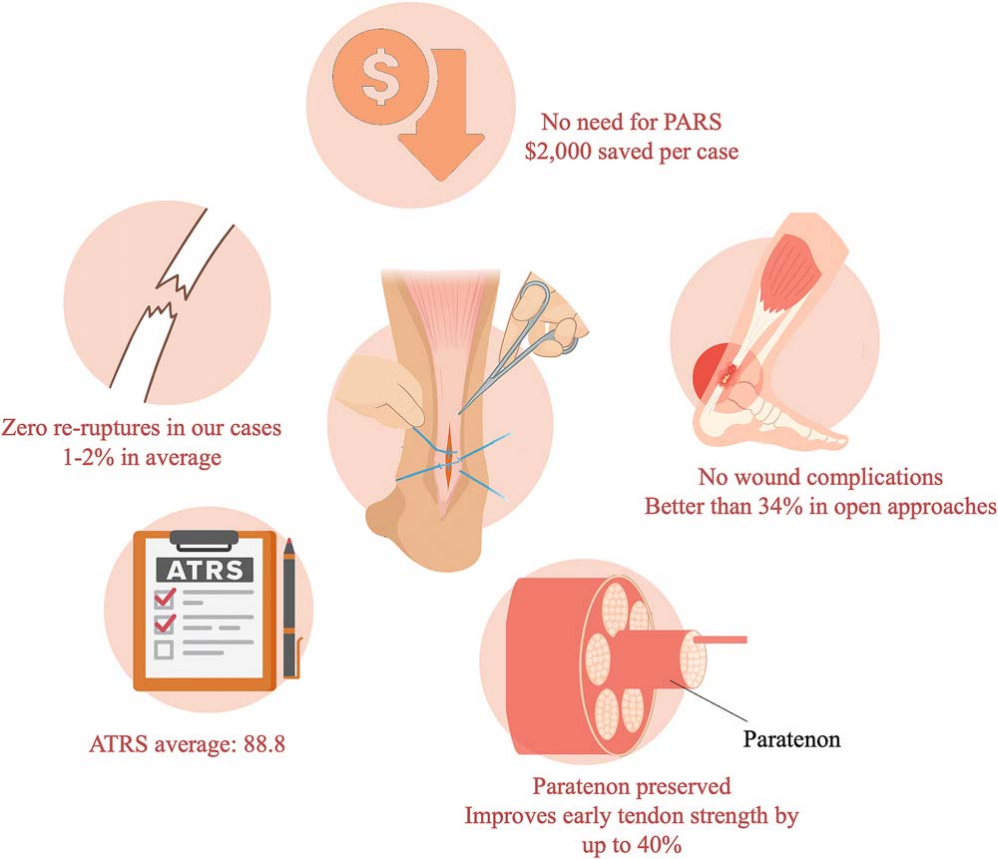
Key outcomes and advantages of the modified limited-open Achilles tendon repair technique.

The technique offers several practical advantages. First, it eliminates reliance on expensive proprietary jigs or implant systems like PARS, resulting in estimated cost savings of over $2000 per case [8], which makes it easily teachable and applicable in a variety of healthcare settings, including resource-limited environments. Second, it avoids common complications associated with traditional open repairs, such as wound infection and nerve injury, by using a limited-access incision with careful tissue handling. Refer to Fig. 7 for more of the illustration data and Table 2 for a summary of comparative findings across ATR approaches.

**Table 2:** Comparison of Achilles tendon repair techniques: Open, PARS, and modified limited-open approach.

Technique	Incision type	Suture type	Tension control	Weight-bearing start	Rerupture rate	Cost
Open Repair	Long midline incision	Nonabsorbable	Manual approximation	6–8 weeks	2%–5%	Low
PARS (percutaneous)	Multiple stab incisions	FiberWire	Limited	3–4 weeks	3%–5%	High
Limited-Open Technique	Lateral 4–6 cm	FiberTape (2 mm)	Sequential tensioning	2–3 weeks	1%–2%	Low

## Future directions

The need for further research is warranted to validate and expand on these findings. For example, Cadaveric biomechanical testing comparing sequential vs. simultaneous suture tying would help define the mechanical advantage of our approach. Prospective randomized controlled trials with larger cohorts and longer follow-up are essential for comparing functional outcomes with existing techniques. Additionally, quantitative assessments using ultrasound elastography, isokinetic strength testing, and functional gait analysis could offer objective measures of repair quality and rehabilitation efficacy. Finally, this concept of tension offloading via sequential suturing may hold potential in repairing other tendons prone to high re-tear rates, such as the rotator cuff or patellar tendon.

## Conclusion

This case series examined the safety, feasibility, and early functional rehabilitation of a modified limited-open ATR technique incorporating sequential suture tensioning. By combining this tension offloading technique with early postoperative rehabilitation using the ASO brace, all patients reported outcomes reflected satisfactory recovery with no reported functionally limiting complications or re-rupture. This technique allowed for controlled healing whilst enabling early weight-bearing by week 2–3.

The overall clinical results were significantly stable, although variations in return to sports and ATRS scores were observed, this was particularly in patients with anatomical comorbidities such as the Haglund deformity. These findings support mechanically secure repair strategies to improve recovery. Larger studies are warranted to validate these outcomes and further improve rehabilitation protocols.
